# Association of changes in histologic severity of nonalcoholic steatohepatitis and changes in patient‐reported quality of life

**DOI:** 10.1002/hep4.2044

**Published:** 2022-07-28

**Authors:** Laura Heath, Paul Aveyard, Jeremy W. Tomlinson, Jeremy F. Cobbold, Dimitrios A. Koutoukidis

**Affiliations:** ^1^ Nuffield Department of Primary Care Health Sciences University of Oxford Oxford UK; ^2^ National Institute for Health Research (NIHR) Oxford Biomedical Research Centre Oxford University Hospitals NHS Foundation Trust Oxford UK; ^3^ Oxford Centre for Diabetes Endocrinology and Metabolism NIHR Oxford Biomedical Research Centre University of Oxford Churchill Hospital Oxford UK; ^4^ Department of Gastroenterology and Hepatology NIHR Oxford Biomedical Research Centre Oxford University Hospitals NHS Foundation Trust John Radcliffe Hospital Oxford UK

## Abstract

Nonalcoholic steatohepatitis (NASH) is a prevalent chronic disease that is associated with a spectrum of liver fibrosis and can lead to cirrhosis. Patients with NASH report lower health‐related quality of life (HRQoL) than the general population. It remains uncertain how changes in histologic severity are associated with changes in HRQoL. This is a secondary analysis of the Farnesoid X Receptor Ligand Obeticholic Acid in NASH Treatment (FLINT) and Pioglitazone, Vitamin E, or Placebo for Nonalcoholic Steatohepatitis (PIVENS) randomized controlled trials in patients with biopsy‐proven NASH. HRQoL was assessed using short form‐36 at baseline and at follow‐up biopsy (at 72 and 96 weeks, respectively). Adjusted linear regression models were used to examine the association between changes in liver fibrosis (primary analysis), nonalcoholic fatty liver disease (NAFLD) activity score (secondary analysis), and changes in HRQoL scores. Compared with stable fibrosis, improvement of fibrosis by at least one stage was significantly associated with improvements only in the physical function component by 1.8 points (95% confidence interval, 0.1, 3.5). Worsening of fibrosis by at least one stage was not associated with statistically significant changes in any HRQoL domain compared with stable fibrosis. Associations between HRQoL and NAFLD disease activity score in the secondary analysis were of similar magnitude. Weight loss was associated with small improvements in physical function, general health, and energy levels. *Conclusion*: Improvements in fibrosis stage were associated with improvements in the physical component of HRQoL, but the clinical impact was modest. As improving fibrosis may not meaningfully improve well‐being, treatment for NASH will be cost effective only if it prevents long‐term hepatic and cardiovascular disease.

## Graphical abstract

People with serious liver disease due to excess body weight typically have lower quality of life than the general population. In this study, we explored whether changes in disease severity is associated with changes in patient reported quality of life from secondary analysis of two randomised controlled trials. We found that some improvements in liver disease were associated with small improvements in patient reported health reported quality of life.

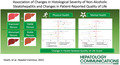

## INTRODUCTION

Nonalcoholic fatty liver disease (NAFLD) is a chronic disease that may progress to nonalcoholic steatohepatitis (NASH), a subtype of NAFLD. The global prevalence of NASH in adults is 1%–6%.^[^ ^1^ ^]^ NASH is strongly associated with obesity and is considered the hepatic component of the metabolic syndrome.^[^ ^2^ ^]^ By 2030, the prevalence of NASH is predicted to rise by 63%, with an associated 137% increase incidence of hepatocellular carcinoma and 168% of decompensated cirrhosis.^[^ ^3^ ^]^ Progression from NAFLD to NASH is associated with an increase in hepatic fibrosis.^[^ ^4^ ^]^ Fibrosis is the factor most strongly associated with long‐term liver morbidity and mortality.^[^ ^5^ ^]^

Patients with NASH have impaired health‐related quality of life (HRQoL) compared with the general population and patients with NAFLD.^[^ ^6^ ^]^ Those with NASH‐related cirrhosis (i.e., fibrosis stage F4) have poorer HRQoL scores than those with noncirrhotic NASH.^[^ ^7^ ^]^ Patients were found to experience a broad range of physical and mental symptoms, especially fatigue, abdominal symptoms, and worry.^[^ ^6^, ^8^ ^]^ Typically, histologic severity is negatively associated with HRQoL in cross‐sectional data.^[^ ^9^ ^]^

There have been numerous trials examining the effectiveness of pharmacological options for NASH, some with modest effects.^[^ ^10^, ^11^, ^12^ ^]^ To date, none of these drugs have been licensed for treatment in Europe or the United States, and the disease is managed mainly by lifestyle modification.^[^ ^13^ ^]^ Understanding the effect pharmacological options have on measures of HRQoL scores can be an important component of the treatment‐approval process.^[^ ^14^ ^]^ Among patients with NASH, data from shorter term trials suggest that improved hepatic fibrosis stage and reduced NAFLD activity score are associated with increased HRQoL scores.^[^ ^15^, ^16^ ^]^ However, research into whether this relationship holds over longer time periods and whether worsening of disease activity is associated with reduced HRQoL scores is limited. The aforementioned trials have also not adjusted for changes in weight, which is a potential significant confounder of such relationships because it is associated with both changes in disease activity and changes in HRQoL.^[^ ^17^, ^18^ ^]^

The Farnesoid X Receptor Ligand Obeticholic Acid in NASH Treatment (FLINT) and Pioglitazone, Vitamin E, or Placebo for Nonalcoholic Steatohepatitis (PIVENS) trials provide an opportunity to investigate this relationship further given their rich data set and primary analysis that showed no change in HRQoL between active treatment and placebo.^[^ ^10^, ^11^ ^]^ These studies provide histologic data from liver biopsies before and after the intervention compared with placebo arms, together with data on weight change and HQRoL. The studies used short form (SF)‐36,^[^ ^19^ ^]^ a validated HRQoL score covering physical and mental components, at the start and end of the trials. The aim of the study was to investigate the association between changes in histologic severity and HRQoL scores in patients with NASH over 1.5 to 2 years, independent of active or placebo treatments and other confounding variables. As the SF‐36 contains information in different HRQoL domains, we investigated whether changes in specific HRQoL areas were associated with changes in histologic severity.

## MATERIALS AND METHODS

### Design and study population

This is a secondary analysis of two published, randomized, controlled trials in adults with NASH: the FLINT^[^ ^10^ ^]^ and PIVENS trials.^[^ ^11^ ^]^ The FLINT trial was a double‐blinded, multicenter, randomized, controlled trial (RCT) investigating 72 weeks of obeticholic acid versus placebo on liver histology for patients with NASH. The PIVENS trial was a double‐blinded, multicenter, three‐armed RCT comparing 96 weeks of treatment with pioglitozone, vitamin E, or placebo on liver histology for patients with NASH but without type 2 diabetes. In the current analysis, we employ a prospective longitudinal design. Ethical approval was granted by University of Oxford, Medical Sciences Division Ethics Committee, reference R74858/RE001, on March 5, 2021.

### Inclusion criteria

All participants in the FLINT and PIVENS trials who had both a baseline and follow‐up evaluable biopsy were included.

### Exclusion criteria

Standard exclusion criteria to trials in NASH applied in the FLINT and PIVENS trials as reported in the original publications^[^ ^10^, ^11^ ^]^ were used in our study.

### Outcomes

The primary outcomes in this analysis were regression coefficients between change in fibrosis stage and the eight components of the SF‐36 score (physical function, physical limitations, pain, general health, energy, social function, emotional limitations, and emotional well‐being). Secondary outcomes were the coefficients between change in the NAFLD disease activity score and the aforementioned eight components of the SF‐36 score. We also investigated regression coefficients between histologic severity (both fibrosis and the NAFLD activity score) and changes in the SF‐36 physical and SF‐36 mental component summary scores.

### Statistical analysis

The analysis followed a prespecified statistical plan (dated January 27, 2021) published ahead of the analysis in the Open Science Framework.^[^ ^20^ ^]^ The primary analysis used linear regression models to explore the association between changes in hepatic fibrosis and changes in HRQoL scores. Changes in fibrosis stage were coded as “improved,” “stable,” or “worsened” if there were a change of ≥−1, 0, or ≥+1 in the stage compared with baseline, respectively, as per the cutoffs for clinically meaningful disease changes in NASH clinical trials.

Following univariable analysis, all models were adjusted for sex (binary), age (continuous), baseline body mass index (BMI) (continuous), baseline fibrosis stage (continuous), baseline value of the HRQoL‐dependent variable in the model, trial (PIVENS or FLINT), treatment (active or placebo), weight change (continuous), and the number of specific comorbidities (scored 0–5 for the presence of type 2 diabetes, gastrointestinal disorders, musculoskeletal or connective tissue disorders, nervous system disorders, and psychiatric disorders).

In the secondary analysis, changes in histologic severity were defined as changes in the NAFLD activity score. This change was coded as “improved,” “stable,” or “worsened” if there were a change of ≥−2, −1 to 1, or ≥+2 in the stage compared with baseline, respectively, as per the cutoffs for clinically meaningful disease changes in NASH clinical trials. The multivariable models were adjusted for the same covariates as the primary analysis with the exception of baseline NAFLD activity score instead of baseline fibrosis stage.

We also investigated changes in summary scores of the physical (physical function, physical role, pain, and general health) and mental (energy, social function, emotional role, and emotional well‐being) components of SF‐36 with histologic severity (both change in fibrosis stage and change in NAFLD activity score). An interaction analysis explored the moderating effect of trial arm (placebo vs. any active treatment) between weight change and NAFLD activity score. Two post hoc exploratory analyses examined whether worsened fibrosis (compared with stable or improved fibrosis) was associated with change in HRQoL and whether improved fibrosis (compared with stable or worsened fibrosis) was associated with change in HRQoL.

When HRQoL data were missing at follow‐up, we employed a last observation carried forward approach because HRQoL was also measured at intermediate time points. If observations were missing at baseline, the next recorded observation was used. Sensitivity analyses on the primary analysis, first, excluded participants with no evidence of fibrosis at both baseline and end of treatment biopsy (as there was no possibility for change) and, second, excluded participants with missing HRQoL data (complete case analysis with no imputation). All analyses were conducted in Stata (version 14.2).

## RESULTS

A total of 421 participants were included in the analysis both from the FLINT (n = 200) and PIVENS (n = 221) trials. Baseline demographics are shown in Table 1, stratified by histologic response. Participants had a mean age of 48.9 (SD, 11.8) years and a mean BMI of 34.3 (SD, 6.5) kg/m^2^; 37.3% of participants were men, 25.4% had a diagnosis of type 2 diabetes, and 54.4% had at least one comorbidity. Participants with worsened fibrosis were of a similar age (*p* = 0.96) and sex (*p* = 0.91) to those with improved fibrosis. There was no evidence that prevalence of type 2 diabetes (*p* = 0.22) or number of comorbidities (*p* = 0.70) differed between worsened or improved fibrosis groups. There was evidence that the percentage of participants with improved and worsened fibrosis differed significantly between trials (*p* = 0.039) and by whether they received an active treatment or placebo (*p* = 0.001).

### HRQoL at baseline

Physical function was significantly lower at baseline among patients with worsened fibrosis at follow‐up compared with those with improved fibrosis (*p* = 0.026; Table 1). However, there was no evidence that other baseline HRQoL scores differed between participants with worsened and improved fibrosis stage or between participants with worsened or improved NAFLD disease activity scores (all *p* > 0.05; Table 1; Table S1).

**TABLE 1:** Baseline characteristics

Factor	Fibrosis Stage			*p* value^b^
	Stable^a^	Worsened^a^	Improved^a^	
n	199	91	131	
Age (years)	49.3 (12.3)	48.5 (11.4)	48.6 (11.4)	0.96
Sex				
Male	75 (37.7%)	34 (37.4%)	48 (36.6%)	0.91
Female	124 (62.3%)	57 (62.6%)	83 (63.4%)	
Number of comorbidities				
0	101 (50.8%)	35 (38.5%)	56 (42.7%)	0.70
1	59 (29.6%)	36 (39.6%)	48 (36.6%)	
2	32 (16.1%)	14 (15.4%)	22 (16.8%)	
3	3 (1.5%)	5 (5.5%)	5 (3.8%)	
4	3 (1.5%)	1 (1.1%)	0 (0.0%)	
5	1 (0.5%)	0 (0.0%)	0 (0.0%)	
Type 2 diabetes	46 (23.1%)	29 (31.9%)	32 (24.4%)	0.22
Trial				
PIVENS	105 (52.8%)	40 (44.0%)	76 (58.0%)	0.039
FLINT	94 (47.2%)	51 (56.0%)	55 (42.0%)	
Active treatment	113 (56.8%)	45 (49.5%)	93 (71.0%)	0.001
BMI (kg/m^2^)	34.2 (6.4)	34.4 (6.0)	34.5 (6.9)	0.95
Weight (kg)	96.9 (21.2)	97.5 (19.9)	97.6 (22.2)	0.99
Baseline fibrosis stage				
0	38 (19.1%)	23 (25.3%)	0 (0.0%)	<0.001
1	63 (31.7%)	35 (38.5%)	41 (31.3%)	
2	41 (20.6%)	21 (23.1%)	50 (38.2%)	
3	53 (26.6%)	12 (13.2%)	38 (29.0%)	
4	4 (2.0%)	0 (0.0%)	2 (1.5%)	
Baseline NAFLD activity				
2	5 (2.5%)	1 (1.1%)	4 (3.1%)	0.014
3	24 (12.1%)	7 (7.7%)	13 (9.9%)	
4	38 (19.1%)	29 (31.9%)	25 (19.1%)	
5	47 (23.6%)	25 (27.5%)	32 (24.4%)	
6	54 (27.1%)	22 (24.2%)	23 (17.6%)	
7	27 (13.6%)	6 (6.6%)	27 (20.6%)	
8	4 (2.0%)	1 (1.1%)	7 (5.3%)	
Physical Function	48.0 (10.0)	45.4 (12.1)	48.6 (9.0)	0.026
Physical Limitations	49.1 (10.7)	47.2 (11.8)	47.9 (10.8)	0.61
Pain	52.1 (9.5)	49.5 (11.9)	50.2 (10.4)	0.61
General health	44.9 (9.0)	41.5 (10.4)	43.8 (9.1)	0.093
Energy	47.7 (9.6)	45.6 (10.4)	47.0 (9.6)	0.28
Social function	49.2 (9.7)	46.9 (11.3)	49.6 (10.0)	0.065
Emotional limitations	49.3 (11.0)	47.8 (12.4)	49.1 (11.0)	0.40
Emotional well‐being	49.0 (10.1)	46.9 (11.1)	47.7 (9.4)	0.58
SF‐36 physical	48.5 (9.7)	45.8 (11.7)	47.8 (9.2)	0.15
SF‐36 mental	48.9 (10.2)	47.3 (10.7)	48.4 (10.5)	0.45

*Note*: Higher HRQoL score indicates better or less frequent symptoms.

Abbreviations: BMI, body mass index; FLINT, Farnesoid X Receptor Ligand Obeticholic Acid in NASH Treatment; NAFLD, nonalcoholic fatty liver disease; PIVENS, Pioglitazone, Vitamin E, or Placebo for Nonalcoholic Steatohepatitis; SF, short form.

^a^ Data show mean (SD) or n (%).

^b^ *p* value comparing improved versus worsened fibrosis stage; *t* test for continuous variables, chi‐squared test for categorical variables.

### Association between change in HRQoL and changes in histologic outcomes

**FIGURE 1:**
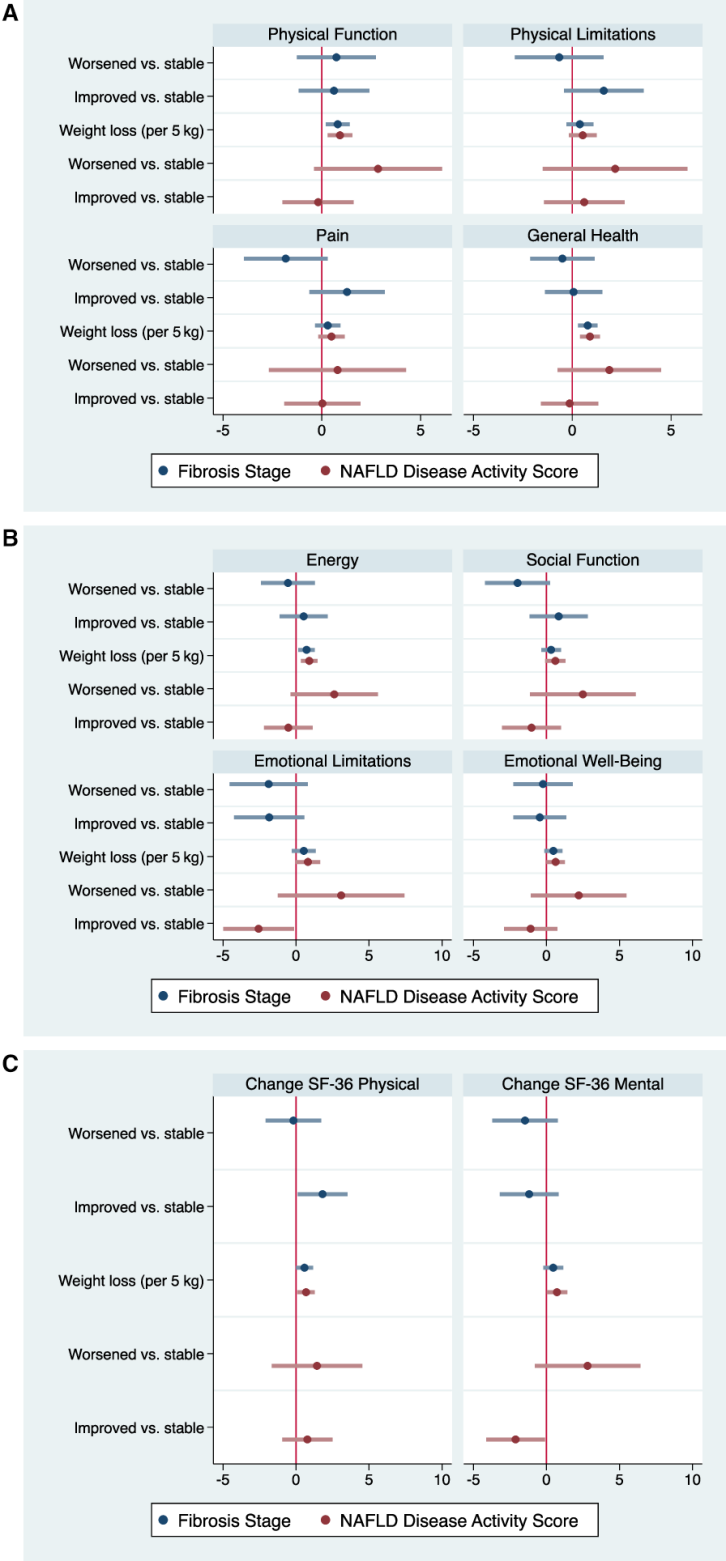
Regression analysis of components of SF‐36. Regression coefficients representing change in (A) SF‐36 physical health component scores, (B) SF‐36 mental health component scores, and (C) SF‐36 physical health and SF‐36 mental health component summary scores, by change in fibrosis stage (worsened vs. stable and improved vs. stable) and NAFLD activity score (worsened vs. stable and improved vs. stable) between baseline and follow‐up in the adjusted models. NAFLD, nonalcoholic fatty liver disease; SF, short form.

Compared with stable disease in adjusted analysis, improved fibrosis was significantly associated with improvements only in the aggregate SF‐36 physical health component of 1.8 (95% confidence interval [CI], 0.1, 3.5) (Figure 1; Table 2). This change was likely driven by cumulative improvements in each of the subdomains of the score, primarily pain and physical limitations, although none of these was statistically significant. In contrast, worsened fibrosis was not associated with statistically significant changes in any HRQoL domain compared with stable disease.

**TABLE 2:** Health‐related quality of life scores over time

	Estimated change in HRQoL score from baseline to follow‐up compared with stable fibrosis or stable NAFLD activity score^a^			
HRQoL domain	Fibrosis improved (95% CI)	Fibrosis worsened (95% CI)	NAFLD activity score improved (95% CI)	NAFLD activity score worsened (95% CI)
Physical function	0.6 (−1.2, 2.4)	0.7 (−1.3, 2.7)	−0.2 (−2.0, 1.6)	2.8 (−0.4, 6.1)
Physical limitations	1.6 (−0.4, 3.6)	−0.7 (−2.9, 1.6)	0.6 (−1.4, 2.6)	2.2 (−1.5, 5.8)
Pain	1.3 (−0.6, 3.2)	−1.8 (−3.9, 0.3)	0.0 (−1.9, 2.0)	0.8 (−2.7, 4.3)
General health	0.1 (−1.4, 1.5)	−0.5 (−2.1, 1.1)	−0.1 (−1.6, 1.3)	1.9 (−0.7, 4.5)
Energy	0.5 (−1.1, 2.2)	−0.6 (−2.4, 1.3)	−0.5 (−2.2, 1.1)	2.6 (−0.4, 5.6)
Social function	0.8 (−1.2, 2.8)	−2.0 (−4.2, 0.2)	−1.0 (−3.0, 1.0)	2.5 (−1.1, 6.1)
Emotional limitations	−1.8 (−4.2, 0.6)	−1.8 (−4.6, 0.8)	−2.6 (−5.0, −0.1)*	3.1 (−1.2, 7.4)
Emotional well‐being	−0.5 (−2.3, 1.4)	−0.2 (−2.3, 1.8)	−1.1 (−2.9, 0.7)	2.2 (−1.1, 5.5)
SF‐36 physical	1.8 (0.1, 3.5)*	−0.2 (−2.1, 1.7)	0.8 (−0.9, 2.5)	1.4 (−1.7, 4.5)
SF‐36 mental	−1.2 (−3.2, 0.8)	−1.5 (−3.7, 0.8)	−2.1 (−4.1, −0.1)*	2.8 (−0.8, 6.4)

*Note:* Higher HRQoL score indicates better or less frequent symptoms.

Abbreviations: CI, confidence interval; HRQoL, health‐related quality of life; NAFLD, nonalcoholic fatty liver disease.

^a^ Adjusted for baseline fibrosis stage/NAFLD activity score, baseline HRQoL score, sex, age, baseline body mass index, study, treatment, weight change, and comorbidities.

* *p* < 0.05.

In the secondary analysis, there was evidence that emotional limitations and aggregate mental health function score worsened by −2.6 (95% CI, –5.0, −0.1) and −2.1 (95% CI, –4.1, −0.1), respectively, with improvements in NAFLD disease activity score. There was no evidence that worsened NAFLD disease activity score was associated with a significant change in any HRQoL domain (Figure 1; Table 2).

**FIGURE 2:**
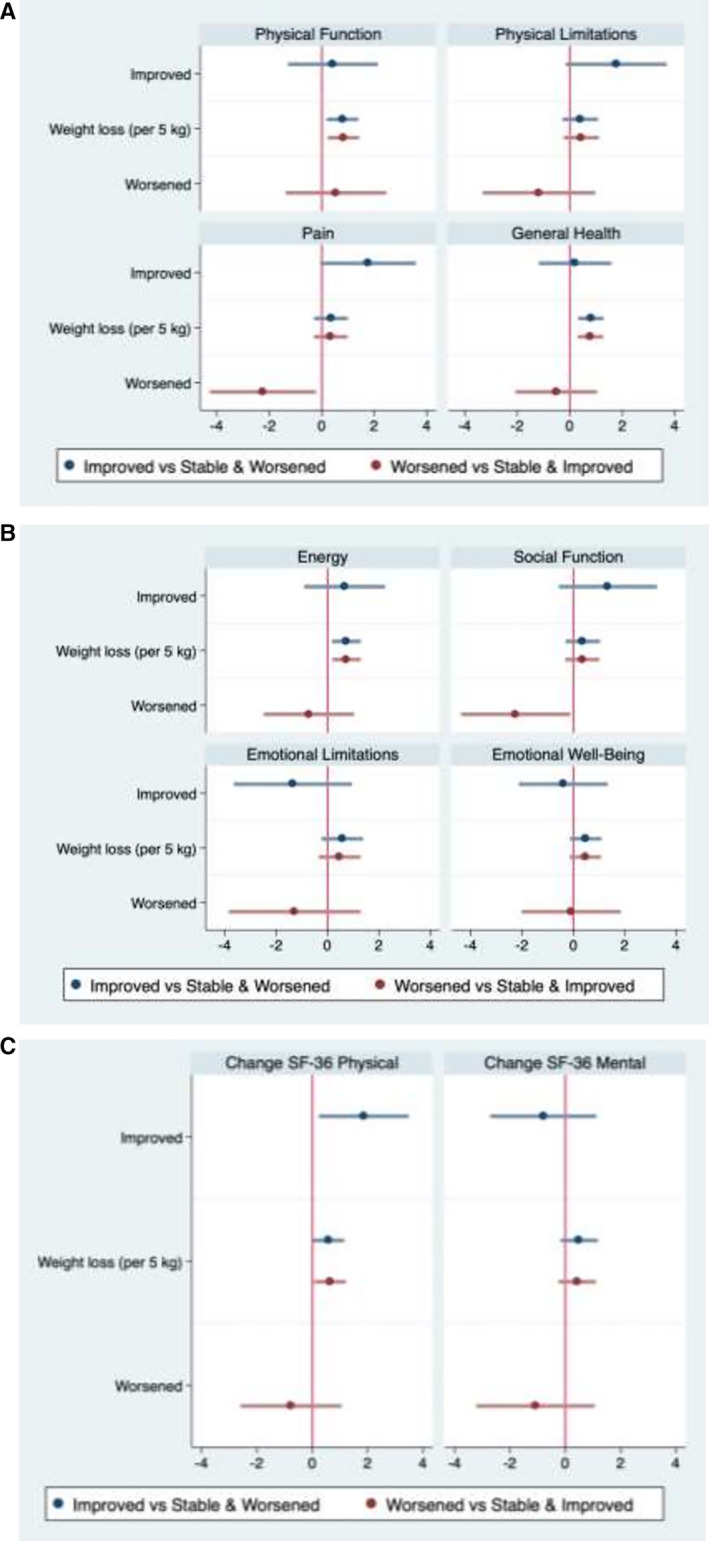
Regression coefficients representing change. (A) SF‐36 physical health component scores, (B) SF‐36 mental health component scores, and (C) SF‐36 physical health and SF‐36 mental health component summary scores, by change in fibrosis stage (improved vs. stable and worsened [regression model in blue]; and worsened vs. stable and improved [regression model in red]) between baseline and follow‐up in the adjusted models. SF, short form.

Baseline demographics, stratified by fibrosis stage as improved versus stable and worsened, and fibrosis stage as worsened versus stable and improved are shown in Tables S2 and S3, respectively. A post hoc exploratory analysis compared improved fibrosis with stable and worsened fibrosis, and another analysis compared worsened fibrosis with stable and improved fibrosis (Figure 2). Results were broadly similar to the primary analysis; improved fibrosis was significantly associated with improved aggregate SF‐36 physical health component by 1.8 points (95% CI, 0.2, 3.5) compared to stable and worsened fibrosis. Worsened fibrosis was additionally significantly associated with worsening of pain score by −2.2 points (95% CI, –4.3, −0.2) and social function score by −2.2 points (95% CI, –4.4, −0.1) compared with stable and improved fibrosis.

### Weight change

In the multivariable model of fibrosis changes, changes in weight, the only modifiable covariate, were associated with HRQoL changes. There was evidence that each 5 kg of weight loss was associated with an increase in physical function of 0.8 points (95% CI, 0.2, 1.4), in general health of 0.8 points (95% CI, 0.3, 1.3), and in energy of 0.7 points (95% CI, 0.2, 1.3). There was no evidence that weight change was associated with the other HRQoL components. The same HRQoL components were significant in the NAFLD disease activity score analysis. There was no evidence of a moderating effect of trial arm (placebo vs. any active treatment) between weight change and NAFLD activity score across all HRQoL domains (*p*_interation_ > 0.05).

### Sensitivity analysis

Sensitivity analysis 1 (Table S4) excluded participants with no evidence of fibrosis at baseline, and end of treatment showed results broadly consistent with the main analysis.

There was evidence that worsened fibrosis was associated with worsened pain and social function. NAFLD disease activity improvements were associated with worsening in aggregate mental health score. Despite these estimates being statistically significant, the point estimates and CIs did not materially differ from the main analysis.

Consistent with the main analysis, complete case analysis (Table S5) found evidence of an association between improvements in the NAFLD score and worsening of emotional limitations and the aggregate SF‐36 mental component score. The association between fibrosis improvements and improvements in the SF‐36 physical component was attenuated, and no other association was statistically significant.

## DISCUSSION

We found some evidence that modest changes in fibrosis stage or the NAFLD activity score were independently associated with statistically significant changes in HRQoL score over 1.5–2 years. The most consistent association was between improvements in fibrosis and improvements in physical function. Our sensitivity and post hoc analyses further suggested that worsening of fibrosis is associated with worsening of pain and social function.

Previous research has suggested that a conservative clinically meaningful change in HRQoL score is half the SD, which in the present study would be approximately 5 points on the SF‐36 scale.^[^ ^21^ ^]^ This would equate to a change of approximately 5 points in the current study, but the observed average change across all scores ranged between −2 and +3, with relatively wide CIs. Although these changes were small and unlikely to be clinically meaningful, they suggest that larger changes in histologic severity, such as from stage 3 fibrosis to stage 0 fibrosis, are necessary to observe meaningful changes in HRQoL. This is in line with analyses showing that patients with NASH cirrhosis have significantly lower HRQoL than those with earlier stage disease.^[^ ^7^ ^]^

Throughout the study, participants had HRQoL scores in all domains below the 1998 US population mean of 50 (SD, 10).^[^ ^22^ ^]^ This supports previous findings that show people with NAFLD and NASH have lower HRQoL scores, in particular, poorer physical HRQoL scores, than the general population.^[^ ^23^, ^24^ ^]^ However, our findings contrast with previous studies that have found significant consistent changes in HRQoL scores with changes in fibrosis stage and NAFLD disease activity score.^[^ ^16^, ^25^ ^]^ These studies did not adjust for weight change, which may be a significant confounder in this relationship. Another analysis found that after adjusting for changes in BMI, fibrosis improvement was associated with improvements in abdominal, emotional, worry, and total components from the Chronic Liver Disease Questionnaire for NASH.^[^ ^25^ ^]^ Consistent with our study, these changes were small and unlikely to translate to clinically meaningful outcomes.

There was some evidence that weight change was independently associated with changes in HRQoL score, notably physical function, general health, and energy. A weight loss of 5 kg was associated with an improvement (increase) of 0.7–0.8 points on the SF‐36 scales for physical function, general health, and energy. This was in line with estimates on the association between changes in BMI and changes in HRQoL from weight‐loss intervention trials in other settings.^[^ ^18^ ^]^ Existing weight‐management support and services offered to patients with NASH lead to an average 3–5‐kg change.^[^ ^26^, ^27^ ^]^ Although this is associated with clinically meaningful improvements in disease activity,^[^ ^17^, ^28^ ^]^ it is unlikely that the weight loss achieved in current care will directly translate to clinically meaningful improvements in HRQoL. Interventions leading to greater weight loss, such as semaglutide,^[^ ^29^ ^]^ may lead to larger HRQoL changes in NASH, as has been shown in a recent analysis.^[^ ^30^ ^]^

These results provide context for regulators, such as the US Food and Drug Administration (FDA) and the European Medicines Agency, while they consider new agents for approval as treatment for NASH. HRQoL data are typically included in the application for approval as worsening of disease symptoms or overall HRQoL might affect the decision‐making process. The FDA has not yet approved obeticholic acid for NASH after considering the balance of potential benefits and risks with the latter, including increases in low‐density lipoprotein cholesterol and pruritus.^[^ ^25^ ^]^ Worsening of symptoms may be reflected on patient‐reported HRQoL measures. Furthermore, regulators require patient‐reported HRQoL measures if they are to be used to support claims in labeling.^[^ ^14^ ^]^ The present study suggests that substantial changes in HRQoL may not be achievable within the medium to long term unless medication leads to prevention of serious complications of NASH, such as severe liver events and cardiovascular disease; longer follow‐up in trials might be necessary to observe meaningful HRQoL changes.

Strengths of this analysis include the preregistered statistical analysis plan, use of a widely validated and reliable measure for HRQoL,^[^ ^31^, ^32^ ^]^ the histologic assessment of disease changes using the benchmark method (i.e., biopsies),^[^ ^33^ ^]^ the blinded and standardized assessment of liver biopsies, and the follow‐up over a 1.5–2‐year period in a well‐defined population with moderately advanced liver disease. The data set was mostly complete, with only seven participants (six in PIVENS and one in FLINT) with missing data in their baseline questionnaire that did not meaningfully affect the estimates in sensitivity analysis. To our knowledge, this was the first analysis to consider the association between worsened (in addition to improved) fibrosis stage or NAFLD disease activity score with HRQoL outcomes.

Limitations include using a general and not disease‐specific HRQoL scale. While this allows comparison of changes in HRQoL scores among other diseases or treatments, the SF‐36 questionnaire may miss some symptoms specific to liver disease, such as abdominal discomfort, that are captured in other validated scales.^[^ ^34^, ^35^ ^]^ It was unclear from the study protocols whether participants completed their SF‐36 before or after the results of their final biopsy were disclosed. If participants were aware of their biopsy results, this could theoretically impact their final HRQoL answers. Although both studies used the SF‐36 questionnaires, there were some subtle differences in the questionnaires used that resulted in a few questions being collapsed to the lowest common denominator. For example, question 18 on the FLINT SF‐36 questionnaire (corresponding to question 19 on PIVENS SF‐36) gave participants five options, whereas PIVENS gave six options. Some detail was lost when the PIVENS answers had to be adjusted to five possible answers. The present analysis also found unexpected associations between worsened mental health HRQoL scores and improved NAFLD activity score. It is unclear why this association was present, and although it was potentially a chance finding, future studies should investigate it further.

In conclusion, we found some evidence that improvement of liver fibrosis stage was associated with statistically but not clinically significant improvements in some HRQoL scores in patients with biopsy‐proven NASH. There was some evidence that weight loss was associated with small improvements of physical function, general health, and energy levels, although again this was not at a magnitude associated with clinically meaningful results. Future trials of potential treatments need to address the longevity and HRQoL improvements that would come with preventing severe complications of NASH, such as liver events and cardiovascular disease.

## FUNDING INFORMATION

This study was funded by the National Institute for Health Research (NIHR) Oxford Biomedical Research Centre (grant number: IS‐BRC‐1215–20008). PA is NIHR Senior Investigator and also funded by the Oxford and Thames Valley NIHR Applied Research Collaboration. LH is an Academic Clinical Fellow funded by the NIHR and received a grant from Guts UK. The funders had no role in the design and conduct of the study; collection, management, analysis, and interpretation of the data; preparation, review, or approval of the manuscript; and decision to submit the manuscript for publication. The views expressed are those of the authors and not necessarily those of the National Health Service, NIHR, or Department of Health and Social Care.

## CONFLICTS OF INTEREST

Dimitrios Koutoukidis, Jeremy Tomlinson, Jeremy Cobbold, and Paul Aveyard have been investigators for the National Institute for Health Research. Paul Aveyard has been an investigator for the Cambridge Weight Plan and a speaker at a conference funded by Novo Nordisk. Jeremy Tomlinson has been part of the scientific advisory boards for Pfizer, Novo Nordisk, and Poxel. Jeremy Cobbold has served on advisory boards and consulted for Intercept, Novo Nordisk, and Alnylam. Laura Heath has nothing to report.

## ETHICS APPROVAL STATEMENT

Ethical approval was granted by the University of Oxford, Medical Sciences Division Ethics Committee, reference R74858/RE001 on March 5, 2021.

## Supporting information


**Appendix S1:** Supporting Information

**Supplementary Table 1.** *Baseline Demographics by NAFLD Disease Activity Score*
NOTE. Higher HRQoL score indicates better or less frequent symptoms
*Figures are mean (SD) or n(%)
**p‐value comparing improved vs worsened fibrosis score; t‐test for continuous variables, Chi‐squared for categorical.
Supplementary Table 2. *Baseline Demographics by Fibrosis Stage (Improved vs Worsened & Stable)*
NOTE. Higher HRQoL score indicates better or less frequent symptoms
*Figures are mean (SD) or n(%)
**p‐value comparing improved vs worsened fibrosis score; t‐test for continuous variables, Chi‐squared for categorical.
Supplementary Table 3. *Baseline Demographics by Fibrosis Stage (Worsened vs Improved & Stable)*
NOTE. Higher HRQoL score indicates better or less frequent symptoms
*Figures are mean (SD) or n(%)
**p‐value comparing improved vs worsened fibrosis score; t‐test for continuous variables, Chi‐squared for categorical. Supplementary Table 4 – Health related quality of life scores over time.
Sensitivity analysis 1: Participants with no evidence of fibrosis at baseline and follow up (n = 38) excluded
NOTE. Higher HRQoL score indicates better or less frequent symptoms
*Adjusted for baseline fibrosis stage/ NAFLD disease activity score, baseline HRQoL score, sex, age, baseline BMI, study, treatment, weight change and comorbidities.
** p < 0.05
Supplementary Table 5– Health related quality of life scores over time.
Sensitivity analysis 2: Complete case analysis
NOTE. Higher HRQoL scores indicates better or less frequent symptoms
*Adjusted for baseline fibrosis stage/NAFLD disease activity score, baseline HRQoL score, sex, age, baseline BMI, study, treatment, weight change and comorbidities.
** p < 0.05
