# Supplementary material for: Association of changes in histologic severity of nonalcoholic steatohepatitis and changes in patient‐reported quality of life
Source: Hepatol Commun. 2022 Jul 28;6(10):2623–33. doi: 10.1002/hep4.2044 (PMC9512481; doi:10.1002/hep4.2044)
Supplement: Supplementary file 1 — Appendix S1: Supporting Information [file HEP4-6-2623-s001.docx]

**Study Title:**  Association of changes in disease activity and changes in patient-reported quality of life in non-alcoholic steatohepatitis (NASH)

**Short title:** Disease activity and quality of life in non-alcoholic steatohepatitis

**MS IDREC Ref:** tbc

**Date and Version No:** 27 January 2021, v2.0

| **Principal Investigator:** | Dr Laura Heath, Academic Clinical Fellow and Dr Dimitrios Koutoukidis, Research Fellow  Nuffield Department of Primary Care Health Sciences, University of Oxford  [dimitrios.koutoukidis@phc.ox.ac.uk](mailto:dimitrios.koutoukidis@phc.ox.ac.uk) +44 01865 617767  laura.heath@phc.ox.ac.uk |
| --- | --- |
| **Investigators:** | Prof Paul Aveyard^1^  Prof Jeremy Tomlinson^1^  Dr Jeremy Cobbold ^1,2^  ^1^University of Oxford  ^2^Oxford University Hospitals NHS Foundation Trust |
| **Sponsor:** | University of Oxford |
| **Funder:** | NIHR Oxford Biomedical Research Centre |
| **Principal Investigator Signature:** | 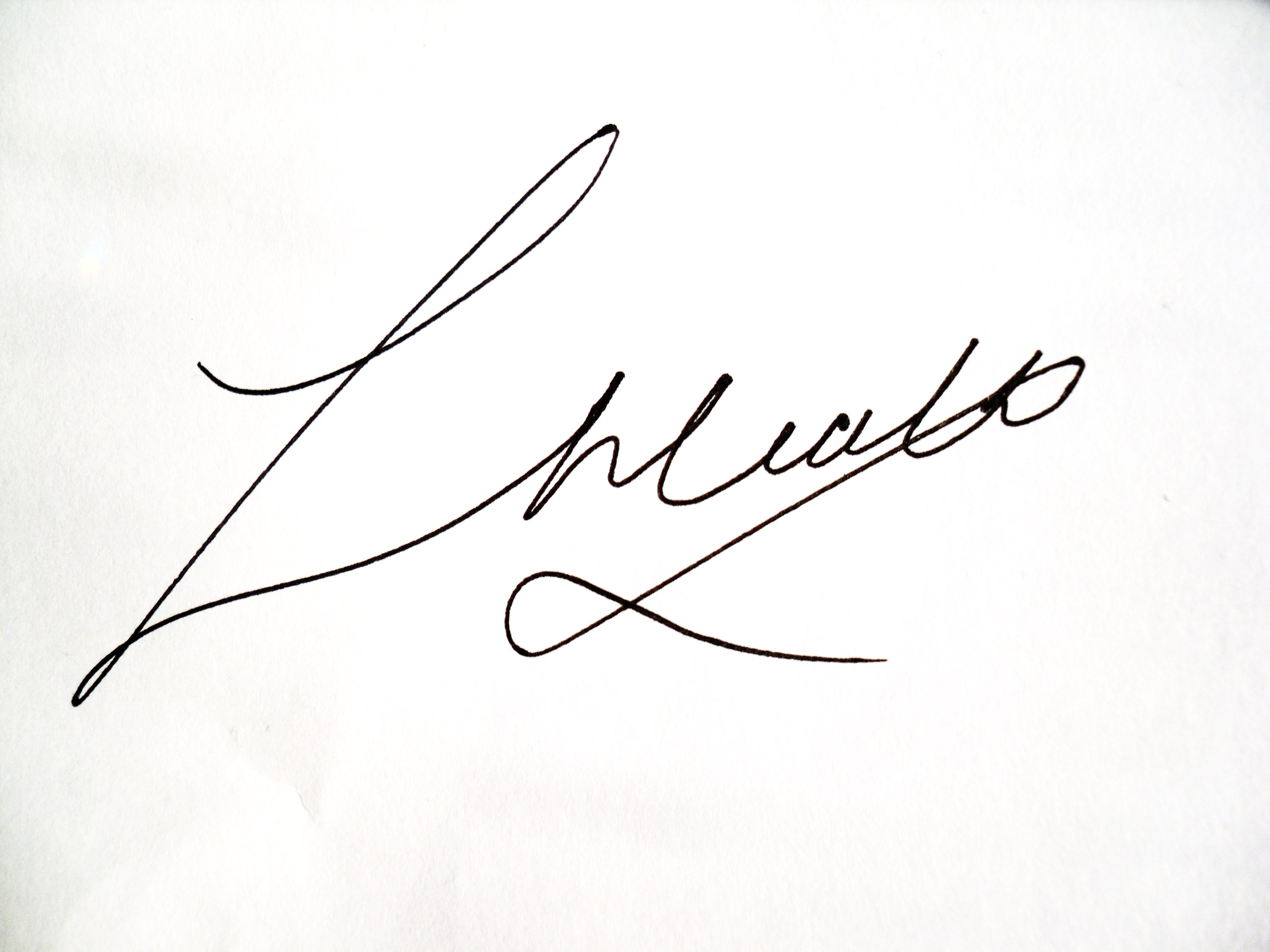 |

**Conflicts of interest**: DK, PA, SJ, JT, JC are investigators in an investigator-initiated trial funded by the NIHR where the weight loss intervention has been donated to participants by Nestle Health Sciences. No other conflicts of interest are reported. PA spoke at symposium at the Royal College of General Practitioners conference that was funded by Novo Nordisk. SAJ attended a one-day meeting on digital health interventions, organised by Oviva. None of these association led to payments to these authors personally. JWT has been part of the scientific advisory board for Pfizer, Novo Nordisk and Poxel. JFC has served on advisory boards and consulted for Intercept, Novo Nordisk and Alnylam.

**Confidentiality Statement:** This document contains confidential information that must not be disclosed to anyone other than the authorised individuals from the University of Oxford, the Investigator Team and members of the Medical Sciences Interdivisional Research Ethics Committee (MS IDREC), unless authorised to do so.

**TABLE OF CONTENTS**

[1. SYNOPSIS 3](#_Toc61549947)

[2. ABBREVIATIONS 3](#_Toc61549948)

[3. BACKGROUND AND RATIONALE 4](#_Toc61549949)

[4. OBJECTIVES AND OUTCOME MEASURES 4](#_Toc61549950)

[5. STUDY DESIGN 5](#_Toc61549951)

[6. PARTICIPANT IDENTIFICATION 5](#_Toc61549952)

[6.1. Study Participants 5](#_Toc61549953)

[6.2. Inclusion Criteria 5](#_Toc61549954)

[6.3. Exclusion Criteria 5](#_Toc61549955)

[7. STATISTICS AND ANALYSIS 6](#_Toc61549956)

[7.1. Analysis of Outcome Measures 6](#_Toc61549957)

[8. DATA MANAGEMENT 7](#_Toc61549958)

[8.1. Access to Data 7](#_Toc61549959)

[8.2. Data Handling and Record Keeping 7](#_Toc61549960)

[9. QUALITY CONTROL AND QUALITY ASSURANCE PROCEDURES 7](#_Toc61549961)

[10. ETHICAL AND REGULATORY CONSIDERATIONS 7](#_Toc61549962)

[10.1. Declaration of Helsinki 7](#_Toc61549963)

[10.2. Approvals 8](#_Toc61549964)

[10.3. Participant Confidentiality 8](#_Toc61549965)

[10.4. Annual Progress Report 8](#_Toc61549966)

[11. FINANCE AND INSURANCE 8](#_Toc61549967)

[11.1. Funding 8](#_Toc61549968)

[11.2. Insurance 8](#_Toc61549969)

[12. PUBLICATION POLICY 8](#_Toc61549970)

[13. REFERENCES 9](#_Toc61549971)

[14. APPENDIX C: AMENDMENT HISTORY 10](#_Toc61549972)

# SYNOPSIS

| **Long Study Title** | Association of changes in disease activity and changes in patient-reported quality of life in non-alcoholic steatohepatitis | |
| --- | --- | --- |
| **Short Study Title** | Disease activity and quality of life in non-alcoholic steatohepatitis | |
| **Nature of Study Participants** | Adults diagnosed with non-alcoholic steatohepatitis who participated in previous clinical trials and agreed for data to be shared | |
| **Intended number of participants** | 422 | |
| **Planned Study Period** | February 2021 – May 2021 | |
|  | **Objectives** | **Outcome Measures** |
| **Primary** | **Primary objectives**  To examine the association between changes in disease activity (fibrosis score) and changes in   1. Physical functioning 2. Physical role 3. Pain 4. General health 5. Fatigue 6. Social function 7. Emotional role 8. Emotional well-being | Univariable and multivariable regression coefficients between changes in disease activity (fibrosis score) and changes in   1. Physical functioning 2. Physical role 3. Pain 4. General health 5. Fatigue 6. Social function 7. Emotional role 8. Emotional well-being |
| **Secondary** | **Secondary objectives**   1. Repeat the primary analysis with the NAFLD activity score as a measure of disease activity 2. To examine the association between changes in disease activity (fibrosis and the NAFLD activity score) and changes in 3. SF-36 physical component 4. SF-36 mental component | 1. Univariable and multivariable regression coefficients between changes in disease activity (as NAFLD activity score) and changes in 2. Physical functioning 3. Physical role 4. Pain 5. General health 6. Fatigue 7. Social function 8. Emotional role 9. Emotional well-being 10. Univariable and multivariable regression coefficients between changes in disease activity (fibrosis and the NAFLD activity score) and changes in 11. SF-36 physical component 12. SF-36 mental component |

# ABBREVIATIONS

| CUREC | Central University Research Ethics Committee |
| --- | --- |
| FLINT | Farnesoid X nuclear receptor ligand obeticholic acid for non-cirrhotic, non-alcoholic steatohepatitis |
| MS IDREC | Medical Sciences Interdivisional Research Ethics Committee |
| NAS | Non-alcoholic fatty liver disease activity score |
| NASH | Non-alcoholic steatohepatitis |
| NIDDK | National Institute of Diabetes and Digestive and Kidney Diseases |
| PIVENS | Pioglitazone versus Vitamin E versus Placebo for the Treatment of Nondiabetic Patients with Non-alcoholic Steatohepatitis |
| PI | Principal Investigator |

# BACKGROUND AND RATIONALE

There have been numerous trials looking at pharmacological options for managing non-alcoholic steatohepatitis (NASH) with modest effects.^1 2^ Yet to date, none of these drugs have been licensed for treatment in Europe and the USA. NASH is strongly associated with obesity, and considered to be the hepatic component of the metabolic syndrome.^3^ NASH is associated with various degrees of hepatic fibrosis, which is the strongest predictor for long-term liver morbidity and mortality. Recent studies have suggested that weight loss is associated with improvements in histological features of NASH and fibrosis,^4^ and intensive weight loss with behavioural support may be an effective treatment option for this group of patients.

Measures of patient-reported health-related quality of life (HRQoL) scores are important parts of decision-making when considering approvals of future treatment options. A recent literature review found that patients with NASH had an impaired HRQoL, compared with the general population, and with NAFLD patients.^5^ Those with NASH-related cirrhosis (i.e. advanced fibrosis) have poorer HRQoL scores than those with non-cirrhotic NASH.^6^ Patients were found to experience a broad range of physical and mental symptoms, especially fatigue, abdominal symptoms, and worry.^5 7^ Typically disease activity is negatively associated with HRQoL in cross-sectional data.^8^

Among patients with NASH, data from a small short-term trial suggest that improvements in hepatic fibrosis and the NAFLD activity score are associated with increased HRQoL scores.^9^ However, it is unclear whether such relationship holds over longer time periods and whether worsening of disease activity is associated with reduced HRQoL scores. The aforementioned trial has also not adjusted changes in weight, which a potential significant confounder of such relationship, because it is associated with both changes in disease activity and changes in HRQoL.

The FLINT and PIVENS trials provide a good opportunity to investigate this relationship further given their rich dataset and primary analysis that showed no change in HRQoL between active treatment and placebo.^1 2^ These studies provide histological data from liver biopsies before and after the intervention compared with placebo arms, together with data on weight change and quality of life using the SF-36^10^ (a validated quality of life score, covering physical and mental components) at the start and end of the trials. The aim of the study is to investigate the association between disease activity and HRQoL scores in patients with NASH over 1.5 to 2 years. As the SF-36 contains information in different QoL domains, we can investigate specific QoL areas that may be impacted by disease activity.

# OBJECTIVES AND OUTCOME MEASURES

| **Objectives** | **Outcome Measures** |
| --- | --- |
| **Primary objectives**  To examine the association between changes in disease activity (fibrosis score) and changes in   1. Physical functioning 2. Physical role 3. Pain 4. General health 5. Fatigue 6. Social function 7. Emotional role 8. Emotional well-being | Univariable and multivariable regression coefficients between changes in disease activity (fibrosis score) and changes in   1. Physical functioning 2. Physical role 3. Pain 4. General health 5. Fatigue 6. Social function 7. Emotional role 8. Emotional well-being |
| **Secondary objectives**   1. Repeat the primary analysis with the NAFLD activity score as a measure of disease activity 2. To examine the association between changes in disease activity (fibrosis and the NAFLD activity score) and changes in 3. SF-36 physical component 4. SF-36 mental component | 1. Univariable and multivariable regression coefficients between changes in disease activity (as NAFLD activity score) and changes in 2. Physical functioning 3. Physical role 4. Pain 5. General health 6. Fatigue 7. Social function 8. Emotional role 9. Emotional well-being 10. Univariable and multivariable regression coefficients between changes in disease activity (fibrosis and the NAFLD activity score) and changes in 11. SF-36 physical component 12. SF-36 mental component |

# STUDY DESIGN

This is a secondary analysis of two previously published randomised controlled trials in adults with NASH: the Farnesoid X nuclear receptor ligand obeticholic acid for non-cirrhotic, non-alcoholic steatohepatitis (FLINT) ^1^ and the Pioglitazone versus Vitamin E versus Placebo for the Treatment of Nondiabetic Patients with Non-alcoholic Steatohepatitis (PIVENS) ^2^. This analysis employs a prospective longitudinal design.

# PARTICIPANT IDENTIFICATION

## Study Participants

Adults (18 years and over) with biopsy-proven NASH. This study will include data from all participants in the FLINT and PIVENS trials who have both a baseline and follow-up evaluable biopsy.

## Inclusion Criteria

- Adults with a biopsy-proven NASH.

## Exclusion Criteria

Standard exclusion criteria to trials in NASH applied in the FLINT and PIVENS trials as previously reported in the original publications ^1 2^.

# STATISTICS AND ANALYSIS

## Analysis of Outcome Measures

Data will be presented as mean (SD) or median (IQR) for continuous data and as percentages for categorical variables for the whole sample.

Correlation coefficients (Pearson’s or Spearman’s as appropriate) will be calculated to explore the univariable relationship between change in disease activity (separately for hepatic fibrosis and the NAFLD activity score) and changes in QoL scores (components of SF-36 as per section 4).

In the primary analysis, linear regression models will be used to explore the association between changes in hepatic fibrosis and changes in QoL scores (components of SF-36 as per section 4). Changes in fibrosis stage will be coded as “improved”, “stable”, “worsened”, if there is a change of >=-1, 0, >=+1 in the stage compared with baseline, respectively, as per the cut-offs for clinically meaningful disease changes in NASH clinical trials. Following univariable analysis, all models will be adjusted for sex (binary), age (continuous), baseline BMI (continuous), baseline fibrosis score (continuous), baseline value of the QoL dependent variable in the model (e.g., fatigue), trial (PIVENS or FLINT) and treatment (active vs. placebo), and weight change (continuous). We will also adjust for a dummy variable (able to take integer values 0-5) representing the number of specific co-morbidities (type II diabetes, GI disorder, MSK/ connective tissue disorders, nervous system disorders and psychiatric disorders)

In a secondary analysis, changes in disease activity will be defined as changes in the NAFLD activity score. This change will be coded as “improved”, “stable”, “worsened”, if there is a change of >=-2, -1–1, >=+2 in the stage compared with baseline, respectively, as per the cut-offs for clinically meaningful disease changes in NASH clinical trials. The multivariable models will be adjusted for the same covariates as the primary analysis with the exception of baseline NAFLD activity score instead of baseline fibrosis score. We will also investigate changes in cumulative totals of the physical (physical functioning, physical role, pain and general health) and mental (fatigue, social function, emotional role and emotional well-being) components of SF-36 with disease activity (both change in fibrosis stage and change in NAFLD activity score).

As SF-36 scores were taken at the same time as the liver biopsy, we anticipate minimal missing SF-36 data. In case of missing HRQoL data at follow-up, we will employ a last observation carried forward approach, since HRQoL was also measured in intermediate time points.

A recent secondary trial analysis (in progress) did not find any evidence of a moderating effect of weight change and change in fibrosis/ NAFLD disease activity score by treatment, trial, or baseline fibrosis score. Therefore, we are not planning to do such moderation analysis. The analysis found evidence of a moderating effect of trial arm (placebo vs. any active treatment) between weight change and NAFLD activity score. We will explore this relationship with the addition of an interaction term; trial arm*weight change.

Sensitivity analysis will be run

1. excluding participants with a diagnosis of no fibrosis at both baseline and end of treatment biopsy
2. complete case analysis (excluding participants with quality-of-life missing data at the biopsy follow up)

There will be no correction for multiple testing and significance will be set at p<0.05.

# DATA MANAGEMENT

## Access to Data

Direct access will be granted to authorised representatives from the University of Oxford for monitoring and/or audit of the study to ensure compliance with regulations.

## Data Handling and Record Keeping

All data (questionnaire, information about health, and physiological test results) are already in the form of electronic files deposited in the Central Repository of the National Institute of Diabetes and Digestive and Kidney Diseases (NIDDK) in the USA. These files will be securely transferred to the University of Oxford using a university-approved mechanism (e.g. OneDrive or equivalent).

We will fully comply with the Human Subject Protection clauses as described in the Data Use Agreement.

The data will be stored on encrypted servers within the University network in a shared drive with limited access only to the study investigators and authorised personnel.

The participants will be identified by a unique study specific number and/or code in any database. The name and any other identifying detail will not be obtained. Data will be analysed in R and STATA.

At the completion of the study, all files containing dataset(s) or any portion thereof will be destroyed and any derivative files and copies shall be destroyed. Destruction of data will follow the standard operating procedures of the departmental information governance guidance. However, we will not have preferential access to the dataset and any researcher can apply to NIDDK to access it in the same way we did.

# QUALITY CONTROL AND QUALITY ASSURANCE PROCEDURES

The study will be conducted in accordance with the current approved protocol, relevant regulations and standard operating procedures.

# ETHICAL AND REGULATORY CONSIDERATIONS

## Declaration of Helsinki

The Investigator will ensure that this study is conducted in accordance with the principles of the Declaration of Helsinki.

## Approvals

The protocol will be submitted to the MS IDREC for written approval.

The Investigator will submit and, where necessary, obtain approval from the above party for all substantial amendments to the original approved documents.

## Participant Confidentiality

As the study will only be obtaining data identified by a study ID but no names or other contact details, there are no issues regarding confidentiality. The study staff will ensure that the participants’ anonymity is maintained. We will fully comply with the Human Subject Protection clauses as described in the Data Use Agreement.

## Annual Progress Report

The PI shall submit on request a Progress Report to the MS IDREC.

# FINANCE AND INSURANCE

## Funding

This study in funded by the NIHR Oxford Biomedical Research Centre.

## Insurance

The University of Oxford has appropriate insurances in place to cover any claim for damages which may arise in connection with this research and for which the University of Oxford is legally liable.

# PUBLICATION POLICY

The Investigators will be involved in reviewing drafts of the manuscripts, abstracts, press releases and any other publications arising from the study. Authors will acknowledge that the study was funded by the NIHR. Authorship will be determined in accordance with the ICMJE guidelines and other contributors will be acknowledged.

# REFERENCES

1. Neuschwander-Tetri BA, Loomba R, Sanyal AJ, et al. Farnesoid X nuclear receptor ligand obeticholic acid for non-cirrhotic, non-alcoholic steatohepatitis (FLINT): a multicentre, randomised, placebo-controlled trial. *Lancet* 2015;385(9972):956-65. doi: 10.1016/S0140-6736(14)61933-4 [published Online First: 2014/11/07]

2. Sanyal AJ, Chalasani N, Kowdley KV, et al. Pioglitazone, vitamin E, or placebo for nonalcoholic steatohepatitis. *N Engl J Med* 2010;362(18):1675-85. doi: 10.1056/NEJMoa0907929 [published Online First: 2010/04/30]

3. Polyzos SA, Kountouras J, Mantzoros CS. Obesity and nonalcoholic fatty liver disease: From pathophysiology to therapeutics. *Metabolism* 2019;92:82-97. doi: 10.1016/j.metabol.2018.11.014 [published Online First: 2018/11/29]

4. Koutoukidis DA KC, Henry JA, Noreik M, Morris E, Manoharan I, et al. The effect of the magnitude of weight loss on non-alcoholic fatty liver disease: a systematic review and meta-analysis. *Under review*

5. Kennedy-Martin T, Bae JP, Paczkowski R, et al. Health-related quality of life burden of nonalcoholic steatohepatitis: a robust pragmatic literature review. *J Patient Rep Outcomes* 2017;2:28. doi: 10.1186/s41687-018-0052-7 [published Online First: 2018/07/10]

6. McSweeney L, Breckons M, Fattakhova G, et al. Health-related quality of life and patient-reported outcome measures in NASH-related cirrhosis. *JHEP Rep* 2020;2(3):100099. doi: 10.1016/j.jhepr.2020.100099 [published Online First: 2020/03/06]

7. Yamamura S, Nakano D, Hashida R, et al. Patient-reported outcomes in patients with non-alcoholic fatty liver disease: A narrative review of Chronic Liver Disease Questionnaire-non-alcoholic fatty liver disease/non-alcoholic steatohepatitis. *J Gastroenterol Hepatol* 2020 doi: 10.1111/jgh.15172 [published Online First: 2020/07/06]

8. Younossi ZM, Stepanova M, Anstee QM, et al. Reduced Patient-Reported Outcome Scores Associate With Level of Fibrosis in Patients With Nonalcoholic Steatohepatitis. *Clin Gastroenterol Hepatol* 2019;17(12):2552-60.e10. doi: 10.1016/j.cgh.2019.02.024 [published Online First: 2019/02/16]

9. Younossi ZM, Stepanova M, Lawitz E, et al. Improvement of hepatic fibrosis and patient-reported outcomes in non-alcoholic steatohepatitis treated with selonsertib. *Liver Int* 2018;38(10):1849-59. doi: 10.1111/liv.13706 [published Online First: 2018/02/22]

10. 36-Item Short Form Survey Instrument (SF-36) <https://www.rand.org/health-care/surveys_tools/mos/36-item-short-form/survey-instrument.html> [

# APPENDIX C: AMENDMENT HISTORY

| **Amendment No.** | **Protocol Version No.** | **Date issued** | **Author(s) of changes** | **Details of Changes made** |
| --- | --- | --- | --- | --- |
|  |  |  |  |  |
