# Supplementary material for: Association of changes in histologic severity of nonalcoholic steatohepatitis and changes in patient‐reported quality of life
Source: Hepatol Commun. 2022 Jul 28;6(10):2623–33. doi: 10.1002/hep4.2044 (PMC9512481; doi:10.1002/hep4.2044)
Supplement: Supplementary file 2 — Supplementary Table 1. Baseline Demographics by NAFLD Disease Activity Score NOTE. Higher HRQoL score indicates better or less frequent symptoms *Figures are mean (SD) or n(%) **p‐value comparing improved vs worsened fibrosis score; t‐test for continuous variables, Chi‐squared for categorical. Supplementary Table 2. Baseline Demographics by Fibrosis Stage (Improved vs Worsened & Stable) NOTE. Higher HRQoL score indicates better or less frequent symptoms *Figures are mean (SD) or n(%) **p‐value comparing improved vs worsened fibrosis score; t‐test for continuous variables, Chi‐squared for categorical. Supplementary Table 3. Baseline Demographics by Fibrosis Stage (Worsened vs Improved & Stable) NOTE. Higher HRQoL score indicates better or less frequent symptoms *Figures are mean (SD) or n(%) **p‐value comparing improved vs worsened fibrosis score; t‐test for continuous variables, Chi‐squared for categorical. Supplementary Table 4 – Health related quality of life scores over time. Sensitivity analysis 1: Participants with no evidence of fibrosis at baseline and follow up (n = 38) excluded NOTE. Higher HRQoL score indicates better or less frequent symptoms *Adjusted for baseline fibrosis stage/ NAFLD disease activity score, baseline HRQoL score, sex, age, baseline BMI, study, treatment, weight change and comorbidities. ** p < 0.05 Supplementary Table 5– Health related quality of life scores over time. Sensitivity analysis 2: Complete case analysis NOTE. Higher HRQoL scores indicates better or less frequent symptoms *Adjusted for baseline fibrosis stage/NAFLD disease activity score, baseline HRQoL score, sex, age, baseline BMI, study, treatment, weight change and comorbidities. ** p < 0.05 [file HEP4-6-2623-s002.docx]

|  |  | NAFLD Disease Activity Score | | |  |
| --- | --- | --- | --- | --- | --- |
| Factor |  | Stable | Worsened | Improved | p-value** |
| n |  | 200 | 28 | 193 |  |
| Age (years) |  | 48.5 (12.0) | 48.5 (13.4) | 49.3 (11.5) | 0.74 |
| Sex | Male | 72 (36.0%) | 15 (53.6%) | 70 (36.3%) | 0.079 |
|  | Female | 128 (64.0%) | 13 (46.4%) | 123 (63.7%) |  |
| Number of comorbidities | 0 | 87 (43.5%) | 9 (32.1%) | 96 (49.7%) | 0.20 |
|  | 1 | 72 (36.0%) | 14 (50.0%) | 57 (29.5%) |  |
|  | 2 | 31 (15.5%) | 5 (17.9%) | 32 (16.6%) |  |
|  | 3 | 6 (3.0%) | 0 (0.0%) | 7 (3.6%) |  |
|  | 4 | 3 (1.5%) | 0 (0.0%) | 1 (0.5%) |  |
|  | 5 | 1 (0.5%) | 0 (0.0%) | 0 (0.0%) |  |
| Type 2 Diabetes | | 52 (26.0%) | 7 (25.0%) | 48 (24.9%) | 0.99 |
| Trial | PIVENS | 96 (48.0%) | 16 (57.1%) | 109 (56.5%) | 0.95 |
|  | FLINT | 104 (52.0%) | 12 (42.9%) | 84 (43.5%) |  |
| Active treatment |  | 95 (47.5%) | 11 (39.3%) | 145 (75.1%) | <0.001 |
| BMI (kg/m^2) | | 35.1 (6.5) | 33.1 (6.8) | 33.8 (6.3) | 0.61 |
| Weight (kg) |  | 98.8 (20.9) | 95.3 (24.5) | 95.9 (21.0) | 0.89 |
| Baseline NAFLD disease activity | 2 | 6 (3.0%) | 2 (7.1%) | 2 (1.0%) | <0.001 |
|  | 3 | 29 (14.5%) | 9 (32.1%) | 6 (3.1%) |  |
|  | 4 | 56 (28.0%) | 13 (46.4%) | 23 (11.9%) |  |
|  | 5 | 52 (26.0%) | 4 (14.3%) | 48 (24.9%) |  |
|  | 6 | 41 (20.5%) | 0 (0.0%) | 58 (30.1%) |  |
|  | 7 | 13 (6.5%) | 0 (0.0%) | 47 (24.4%) |  |
|  | 8 | 3 (1.5%) | 0 (0.0%) | 9 (4.7%) |  |
| Baseline fibrosis stage | 0 | 35 (17.5%) | 4 (14.3%) | 22 (11.4%) | 0.28 |
|  | 1 | 57 (28.5%) | 15 (53.6%) | 67 (34.7%) |  |
|  | 2 | 56 (28.0%) | 4 (14.3%) | 52 (26.9%) |  |
|  | 3 | 49 (24.5%) | 5 (17.9%) | 49 (25.4%) |  |
|  | 4 | 3 (1.5%) | 0 (0.0%) | 3 (1.6%) |  |
| Physical Function | | 46.4 (10.8) | 50.2 (8.4) | 48.6 (9.8) | 0.40 |
| Physical Limitations | | 47.5 (11.3) | 49.9 (10.1) | 48.9 (10.8) | 0.63 |
| Pain |  | 49.6 (10.7) | 54.7 (7.9) | 51.8 (10.2) | 0.15 |
| General Health | | 42.9 (10.3) | 46.4 (7.6) | 44.4 (8.7) | 0.25 |
| Energy |  | 45.9 (10.4) | 49.8 (10.1) | 47.8 (9.0) | 0.28 |
| Social Function | | 48.0 (10.9) | 52.1 (7.4) | 49.2 (9.7) | 0.13 |
| Emotional Limitations | | 48.5 (11.6) | 48.2 (11.8) | 49.4 (11.0) | 0.58 |
| Emotional Well-being | | 48.3 (10.5) | 50.7 (10.4) | 47.6 (9.6) | 0.12 |
| SF-36 Physical | | 46.3 (10.7) | 50.8 (7.9) | 48.7 (9.5) | 0.27 |
| SF-36 Mental | | 48.3 (10.7) | 49.6 (10.9) | 48.2 (10.0) | 0.50 |

Supplementary Table 1

*Baseline Demographics by NAFLD Disease Activity Score*

NOTE. Higher HRQoL score indicates better or less frequent symptoms

*Figures are mean (SD) or n(%)

**p-value comparing improved vs worsened fibrosis score; t-test for continuous variables, Chi-squared for categorical.

|  |  | Fibrosis Stage | |  |
| --- | --- | --- | --- | --- |
| Factor |  | Improved | Worsened &Stable | p-value** |
| n |  | 131 | 290 |  |
| Age (years) |  | 48.6 (11.4) | 49.0 (12.0) | 0.73 |
| Sex | Male | 48 (36.6%) | 109 (37.6%) | 0.85 |
|  | Female | 83 (63.4%) | 181 (62.4%) |  |
| Number of comorbidities | 0 | 56 (42.7%) | 136 (46.9%) | 0.64 |
|  | 1 | 48 (36.6) | 95 (32.8%) |  |
|  | 2 | 22 (16.8%) | 46 (15.9%) |  |
|  | 3 | 5 (3.8%) | 8 (2.8%) |  |
|  | 4 | 0 (0.0%) | 4 (1.4%) |  |
|  | 5 | 0 (0.0%) | 1 (0.3%) |  |
| Type 2 Diabetes | | 32 (24.4%) | 75 (25.9%) | 0.75 |
| Trial | PIVENS | 76 (58.0%) | 145 (50.0%) | 0.13 |
|  | FLINT | 55 (42.0%) | 145 (50.0%) |  |
| Active treatment |  | 93 (71.0%) | 158 (54.5%) | 0.001 |
| BMI (kg/m^2) | | 34.5 (6.9) | 34.3 (6.3) | 0.80 |
| Weight (kg) |  | 97.6 (22.2) | 97.1 (20.8) | 0.84 |
| Baseline NAFLD disease activity | 2 | 4 (3.1%) | 6 (2.1%) | 0.036 |
|  | 3 | 13 (9.9%) | 31 (10.7%) |  |
|  | 4 | 25 (19.1%) | 67 (23.1%) |  |
|  | 5 | 32 (24.4%) | 72 (24.8%) |  |
|  | 6 | 23 (17.6%) | 76 (26.2%) |  |
|  | 7 | 27 (20.6%) | 33 (11.4%) |  |
|  | 8 | 7 (5.3%) | 5 (1.7%) |  |
| Baseline fibrosis stage | 0 | 0 (0.0%) | 61 (21.0%) | <0.001 |
|  | 1 | 41 (31.3%) | 98 (33.8%) |  |
|  | 2 | 50 (38.2%) | 62 (21.4%) |  |
|  | 3 | 38 (29.0%) | 65 (22.4%) |  |
|  | 4 | 2 (1.5%) | 4 (1.4%) |  |
| Physical Function | | 48.6 (9.0) | 47.2 (10.7) | 0.19 |
| Physical Limitations | | 47.9 (10.8) | 48.5 (11.1) | 0.64 |
| Pain |  | 50.2 (10.4) | 51.3 (10.4) | 0.34 |
| General Health | | 43.8 (9.1) | 43.9 (9.6) | 0.94 |
| Energy |  | 47.0 (9.6) | 47.0 (9.9) | 0.97 |
| Social Function | | 49.6 (10.0) | 48.5 (10.3) | 0.32 |
| Emotional Limitations | | 49.1 (11.0) | 48.8 (11.5) | 0.79 |
| Emotional Well-being | | 47.7 (9.4) | 48.3 (10.5) | 0.56 |
| SF-36 Physical | | 47.8 (9.2) | 47.7 (10.4) | 0.90 |
| SF-36 Mental | | 48.4 (10.5) | 48.4 (10.3) | 0.98 |

Supplementary Table 2

*Baseline Demographics by Fibrosis Stage (Improved vs Worsened & Stable)*

NOTE. Higher HRQoL score indicates better or less frequent symptoms

*Figures are mean (SD) or n(%)

**p-value comparing improved vs worsened fibrosis score; t-test for continuous variables, Chi-squared for categorical.

Supplementary Table 3

*Baseline Demographics by Fibrosis Stage (Worsened vs Improved & Stable)*

NOTE. Higher HRQoL score indicates better or less frequent symptoms

|  |  | Fibrosis Stage | |  |
| --- | --- | --- | --- | --- |
| Factor |  | Worsened | Improved  &Stable | p-value** |
| n |  | 91 | 330 |  |
| Age (years) |  | 48.5 (11.4) | 49.0 (12.0) | 0.73 |
| Sex | Male | 34 (37.4%) | 123 (37.3%) | 0.99 |
|  | Female | 57 (62.6%) | 207 (62.7%) |  |
| Number of comorbidities | 0 | 35 (38.5%) | 157 (47.6%) | 0.43 |
|  | 1 | 36 (39.6%) | 107 (32.4%) |  |
|  | 2 | 14 (15.4%) | 54 (16.4%) |  |
|  | 3 | 5 (5.5%) | 8 (2.4%) |  |
|  | 4 | 1 (1.1%) | 3 (0.9%) |  |
|  | 5 | 0 (0.0%) | 1 (0.3%) |  |
| Type 2 Diabetes | | 29 (31.9%) | 78 (23.6%) | 0.11 |
| Trial | PIVENS | 40 (44.0%) | 181 (54.8%) | 0.065 |
|  | FLINT | 51 (56.0%) | 149 (45.2%) |  |
| Active treatment |  | 45 (49.5%) | 206 (45.2%) | 0.026 |
| BMI (kg/m^2) | | 34.4 (6.0) | 34.3 (6.6) | 0.92 |
| Weight (kg) |  | 97.5 (19.9) | 97.2 (21.6) | 0.89 |
| Baseline NAFLD disease activity | 2 | 1 (1.1%) | 9 (2.7%) | 0.037 |
|  | 3 | 7 (7.7%) | 37 (11.2%) |  |
|  | 4 | 29 (31.9%) | 63 (19.1%) |  |
|  | 5 | 25 (27.5%) | 79 (23.9%) |  |
|  | 6 | 22 (24.2%) | 77 (23.3%) |  |
|  | 7 | 6 (6.6%) | 54 (16.4%) |  |
|  | 8 | 1 (1.1%) | 11 (3.3%) |  |
| Baseline fibrosis stage | 0 | 23 (25.3%) | 38 (11.5%) | <0.001 |
|  | 1 | 35 (38.5%) | 104 (31.5%) |  |
|  | 2 | 21 (23.1%) | 91 (27.6%) |  |
|  | 3 | 12 (13.2%) | 91 (27.6%) |  |
|  | 4 | 0 (0.0%) | 6 (1.8%) |  |
| Physical Function | | 45.4 (12.1) | 48.2 (9.6) | 0.02 |
| Physical Limitations | | 47.2 (11.8) | 48.6 (10.7) | 0.26 |
| Pain |  | 49.5 (11.9) | 51.4 (9.9) | 0.12 |
| General Health | | 41.5 (10.4) | 44.5 (9.1) | 0.009 |
| Energy |  | 45.6 (10.4) | 47.4 (9.6) | 0.11 |
| Social Function | | 46.9 (11.3) | 49.4 (9.8) | 0.04 |
| Emotional Limitations | | 47.8 (12.4) | 49.2 (11.0) | 0.29 |
| Emotional Well-being | | 46.9 (11.1) | 48.5 (9.8) | 0.20 |
| SF-36 Physical | | 45.8 (11.7) | 48.2 (9.5) | 0.039 |
| SF-36 Mental | | 47.3 (10.7) | 48.7 (10.3) | 0.25 |

*Figures are mean (SD) or n(%)

**p-value comparing improved vs worsened fibrosis score; t-test for continuous variables, Chi-squared for categorical.

Supplementary Table 4 – Health related quality of life scores over time.

*Sensitivity analysis 1: Participants with no evidence of fibrosis at baseline and follow up (n=38) excluded*

|  |  |  | Estimated change in HRQoL score from baseline to follow up* | | | |
| --- | --- | --- | --- | --- | --- | --- |
| HRQoL domain | Baseline score  (mean, SD) | Follow up score  (mean, SD) | Fibrosis improved  (95% CI) | Fibrosis worsened  (95% CI) | NAFLD improved  (95% CI) | NAFLD worsened  (95% CI) |
| Physical Function | 47.4 (10.2) | 46.3 (10.8) | 0.4 (-1.4, 2.2) | 0.3 (-1.9, 2.4) | 0.0 (-1.9, 1.9) | 2.7 (-0.6, 6.1) |
| Physical Limitations | 48.1 (11.0) | 47.5 (11.5) | 1.5 (-0.6, 3.6) | -1.0 (-3.4, 1.5) | 1.0 (-1.2, 3.1) | 2.2 (-1.6, 6.1) |
| Pain | 50.8 (10.4) | 50.2 (10.4) | 1.0 (-0.9, 3.0) | -2.4 (-4.6, -0.1)** | 0.4 (-1.6, 2.4) | 0.3 (-3.2, 3.9) |
| General Health | 43.5 (9.4) | 44.2 (9.7) | 0.1 (-1.4, 1.6) | -0.4 (-2.2, 1.3) | -0.1 (-1.6, 1.4) | 2.0 (-0.7, 4.6) |
| Energy | 46.8 (9.8) | 47.4 (9.9) | 0.5 (-1.2, 2.2) | -0.7 (-2.7, 1.2) | -0.5 (-2.2, 1.2) | 2.1 (-0.9, 5.1) |
| Social Function | 48.8 (10.2) | 47.0 (11.5) | 1.0 (-1.1, 3.1) | -1.8 (-4.3, -0.6)** | -0.7 (-2.9, 1.4) | 2.8 (-1.0, 6.6) |
| Emotional Limitations | 48.9 (11.4) | 48.2 (12.0) | -2.0 (-4.5, 0.6) | -2.0 (-4.9, 1.0) | -2.4 (-5.0, 0.1) | 3.5 (-1.0, 8.0) |
| Emotional Well-being | 47.9 (10.2) | 47.8 (10.8) | -0.3 (-2.2, 1.6) | 0.0 (-2.2, 2.2) | -1.3 (-3.2, 0.6) | 2.1 (-1.3, 5.6) |
| SF-36 Physical | 47.4 (9.8) | 47.0 (10.5) | 1.6 (-0.1, 3.3) | -0.7 (-2.7, 1.3) | 1.2 (-0.6, 2.9) | 1.2 (-1.9, 4.4) |
| SF-36 Mental | 48.3 (10.6) | 47.9 (11.3) | -1.0 (-3.1, 1.1) | -1.1 (-3.6, 1.3) | -2.2 (-4.3, -0.1)** | 2.9 (-0.8, 6.7) |

NOTE. Higher HRQoL score indicates better or less frequent symptoms

*Adjusted for baseline fibrosis stage/ NAFLD disease activity score, baseline HRQoL score, sex, age, baseline BMI, study, treatment, weight change and comorbidities.

** p<0.05

|  |  |  | Estimated change in HRQoL score from baseline to follow up* | | | |
| --- | --- | --- | --- | --- | --- | --- |
| HRQoL domain | Baseline score  (mean, SD) | Follow up score  (mean, SD) | Fibrosis improved  (95% CI) | Fibrosis worsened  (95% CI) | NAFLD improved  (95% CI) | NAFLD worsened  (95% CI) |
| Physical Function | 47.7 (10.3) | 46.5 (10.9) | 0.9 (-0.9, 2.7) | 0.8 (-1.3, 2.8) | -0.3 (-2.1, 1.6) | 2.6 (-0.7, 6.0) |
| Physical Limitations | 48.3 (11.0) | 47.7 (11.4) | 1.6 (-0.4, 3.6) | -0.7 (-2.9, 1.6) | 0.6 (-1.4, 2.6) | 2.2 (-1.5, 5.9) |
| Pain | 51.0 (10.4) | 50.4 (10.6) | 1.3 (-0.6, 3.2) | -1.7 (-3.9, 0.4) | 0.0 (-1.9, 1.9) | 1.2 (-2.3, 4.7) |
| General Health | 43.8 (9.4) | 44.5 (9.7) | 0.1 (-1.4, 1.5) | -0.5 (-2.2, 1.1) | -0.1 (-1.6, 1.3) | 1.9 (-0.8, 4.6) |
| Energy | 47.1 (9.8) | 47.6 (10.1) | 0.4 (-1.2, 2.1) | -0.9 (-2.8, 1.0) | -0.6 (-2.3, 1.1) | 2.5 (-0.5, 5.6) |
| Social Function | 48.8 (10.2) | 47.2 (11.4) | 0.8 (-1.2, 2.8) | -2.1 (-4.3, 0.1) | -0.9 (-2.9, 1.1) | 2.4 (-1.3, 6.1) |
| Emotional Limitations | 48.9 (11.3) | 48.5 (11.8) | -1.9 (-4.3, 0.5) | -2.0 (-4.7, 0.7) | -2.5 (-4.9, -0.1)** | 3.1 (-1.6, 7.5) |
| Emotional Well-being | 48.1 (10.1) | 47.9 (10.6) | -0.4 (-2.2, 1.5) | -0.3 (-2.4, 1.7) | -0.9 (-2.8, 0.9) | 2.1 (-1.3, 5.5) |
| SF-36 Physical | 47.7 (10.1) | 47.2 (10.7) | 1.7 (0.0, 3.5) | -0.1 (-2.0, 1.9) | 0.5 (-1.3, 2.3) | 1.5 (-1.7, 4.8) |
| SF-36 Mental | 48.4 (10.4) | 48.2 (11.0) | -1.4 (-3.5, 0.6) | -1.9 (-4.1, 0.4) | -2.2 (-4.3, -0.2)** | 2.9 (-0.9, 6.7) |

Supplementary Table 5– Health related quality of life scores over time.

*Sensitivity analysis 2: Complete case analysis*

NOTE. Higher HRQoL scores indicates better or less frequent symptoms

*Adjusted for baseline fibrosis stage/NAFLD disease activity score, baseline HRQoL score, sex, age, baseline BMI, study, treatment, weight change and comorbidities.

** p<0.05
